# Effects of exercise therapy on anxiety and depression in patients with COVID-19: a systematic review and meta-analysis

**DOI:** 10.3389/fpubh.2024.1330521

**Published:** 2024-03-06

**Authors:** Ju Tang, Liang-Liang Chen, Hongtao Zhang, Peifeng Wei, Feng Miao

**Affiliations:** ^1^Shaanxi University of Chinese Medicine, Xianyang, China; ^2^The Second Affiliated Hospital of Shaanxi University of Chinese Medicine, Xianyang, China

**Keywords:** exercise therapy, COVID-19, anxiety, depression, meta-analysis

## Abstract

**Objective:**

With increasing rates of anxiety and depression during COVID-19, exercise treatment has drawn attention for its effects on COVID-19 patients with anxiety and depression. This study set out to assess the impact of exercise therapy on COVID-19 patients’ anxiety and depression.

**Methods:**

PubMed, EMBASE, Web of Science and Cochrane Library were used to search articles about exercise therapy as a means of treating anxiety and depression in COVID-19 patients from inception to April 30, 2023. The risk of bias was assessed by the Cochrane Collaboration bias risk tool. Data were pooled with the random effects model. RevMan version 5.4 was used for the statistical analyses. This work was registered in the PROSPERO database (registration number: CRD42023406439).

**Selection criteria:**

Randomized clinical trials (RCTs) of COVID-19 patients with anxiety and depression were included to assess the impact of physical exercise on COVID-19 patients with anxiety and depression.

**Results:**

6 studies including a total of 461 COVID-19 patients were analyzed in this meta-analysis. Overall, the meta-analysis showed that compared with the control group, exercise could significantly improve anxiety (SMD = −0.76; 95%CI: −0.96, −0.55; *p* < 0.00001), depression level (SMD = −0.39; 95%CI: −0.70, −0.09; *p* = 0.01), the PHQ-9 score (MD = −1.82; 95%CI: −2.93, −0.71; *p* = 0.001) and the sleep quality (SMD = −0.73; 95%CI: −1.32, −0.14; *p* = 0.01) in COVID-19 patients.

**Conclusion:**

The research provided evidence that exercise therapy is able to help COVID-19 patients experience less anxiety and depression and have better-quality sleep.

**Systematic review registration:**

CRD42023406439.

## Introduction

The novel coronavirus SARS-CoV-2 caused a febrile respiratory sickness outbreak, which swiftly led to a global outbreak known as coronavirus disease 2019 (COVID-19) (1, 2). The WHO estimates that there are currently over 760 million infected COVID-19 and over 6 million fatalities worldwide (3). To date, there is no specific therapy established to treat COVID-19 and only symptomatic treatment can alleviate the symptoms, but it is unable to keep up with the rate of mutation (4). In particular, the appearance of the Omicron variant BA.1 has presented a rigorous challenge in the fight against COVID-19 (5). COVID-19 in addition to seriously jeopardizing the physical and mental health of COVID-19 patients, it caused severe morbidity, mortality, and financial stress for families and society (6).

Both depression and anxiety are severe neurological illnesses that may lead to suicidal thoughts and self-inflicted injury, as well as impairing memory and sleep. Globally, the COVID-19 epidemic has a profound psychological impact. Studies have shown that patients with COVID-19 had a markedly elevated chance of acquiring mental health issues, especially anxiety and depression. The total prevalence of anxiety and the total prevalence of depression were over 40% (7–9). Despite the fact that there are currently effective medications for depression or anxiety, many individuals do not benefit from them, find them intolerable, or get withdrawal symptoms when the medication is stopped (10). In recent years, many people who suffer from depression or anxiety turn to unconventional and non-pharmacological therapies (11). A randomized controlled trial showed that young people hospitalized to mental hospitals for anxiety and depression benefit both physically and psychologically from physical exercise (12). Exercise may offer individuals with COVID-19 and anxiety and depression a flexible, easy-to-use, and promising therapeutic option. The clinical evidence hierarchy is headed by a systematic review and meta-analysis (13). For the purpose of providing a reference for treating health crises brought on by post-COVID-19 anxiety and depression, this study will carry out a meta-analysis and systematic review to look into the impacts of exercise therapy in treating anxiety and depression produced by COVID-19.

## Methods

The Preferred Reporting Items for Systematic Reviews and Meta-analyses (PRISMA) criteria were followed in the conduct of this meta-analysis (14). And the PRISMA-Checklist is added in the Supplement.

### Search strategy

This study searched articles about RCTs that the impacts of exercise therapy on COVID-19 patients with anxiety and depression in the online databases of PubMed, EMBASE, Web of Science and Cochrane Library from inception to April 30, 2023. We devised search techniques that blended free-text phrases containing people with COVID-19, depression, anxiety, exercise treatment, and randomized clinical trials (RCTs) with medical subject categories. The search strategy used in each database is shown in Supplementary File S1.

### Inclusion criteria and exclusion criteria

**Table:** 

Inclusion criteria
- Study population: COVID-19 patients who have symptoms of anxiety and depression
- Study type: RCTs
- Primary outcome indicators: the degree of anxiety and depression
- Secondary outcome indicators: Patient Health Questionnaire-9 (PHQ-9) and Quality of sleep
- Published from inception to April 30, 2023
Exclusion criteria
- Overview, review, protocol
- Meta-analysis
- Non-RCTs
- RCTs without published outcome indicators
- Non-COVID-19 patients, non-anxiety or non-depression
- Non-exercise therapy
- Letter, comment, abstract, chapter, erratum, dissertation or editorial journal

### Interventions

Patients in the experimental group were treated by exercise therapy. The comparison groups involve other treatments except exercise therapy.

### Outcome

The main results were the degree of anxiety and depression, with scores changing during treatment. Anxiety was assessed by the Self-Rating Anxiety Scale (SAS), the Hamilton Anxiety Rating Scale (HAM-A), State Anxiety Inventory (SAI), Generalized Anxiety Disorder Scale-7 Item (GAD-7), Hospital Anxiety and Depression Scale (HADS). Depression was assessed by the HADS, Self-Rating Depression Scale (SDS), Beck Depression Inventory (BDI). Secondary outcomes were Patient Health Questionnaire-9 (PHQ-9) and Quality of sleep.

### Study selection

The studies are independently reviewed and screened by two reviewers (JT and HZ) in accordance with the review’s inclusion and exclusion criteria. To exclude duplicate articles, the reviewers used EndNote X9 software. They then study the article titles and abstracts to weed out any plainly irrelevant material. They next read the complete contents of the remaining publications, filter them, and justify the exclusion of the researches that were not qualified. Disputes were resolved through discussing with the third reviewer (L-LC).

### Data collection and analysis

Independently, after reading the literature, two reviewers (PW and L-LC) took the data out of the studies that were included. Each study’s details, such as the publication year, first author, sample size, participant age, intervention, control, treatment plan, and primary outcome, were among the data that were gathered. In cases where there was a disagreement between the two reviewers throughout the screening process, the judgment was ultimately made after consulting a third reviewer (FM).

### Risk of bias analysis of included studies

Two researchers (PW and L-LC) used the seven-item Cochrane Collaboration Risk of Bias assessment to independently assess the methodological quality of the literature (15). Each component’s bias risk was evaluated, and the findings were divided into three risk categories: low risk of bias, unclear risk of bias and high risk of bias and then the risk of bias assessment was plotted by Review Manager 5.4 software.

### Data analysis

RevMan version 5.4 was used to analyze the statistical. Continuous outcomes were analyzed using MD or SMD with a 95% CI, and the SMD statistic was selected when the outcome was assessed using different scales. Data were pooled with the random effects model. The assessment of study heterogeneity was conducted using *I*^2^ statistic measurements. If *I*^2^ < 25%, will be considered as no significant heterogeneity. *I*^2^ is between 25 and 50%, will be considered as moderate heterogeneity. *I*^2^ > 50% will be considered as significant heterogeneity. When *I*^2^ ≥ 25%, subgroup analysis was performed to identify the source of any clinical heterogeneity seen in the pooled findings.

## Results

### Study selection and basic characteristics

**Figure 1:**
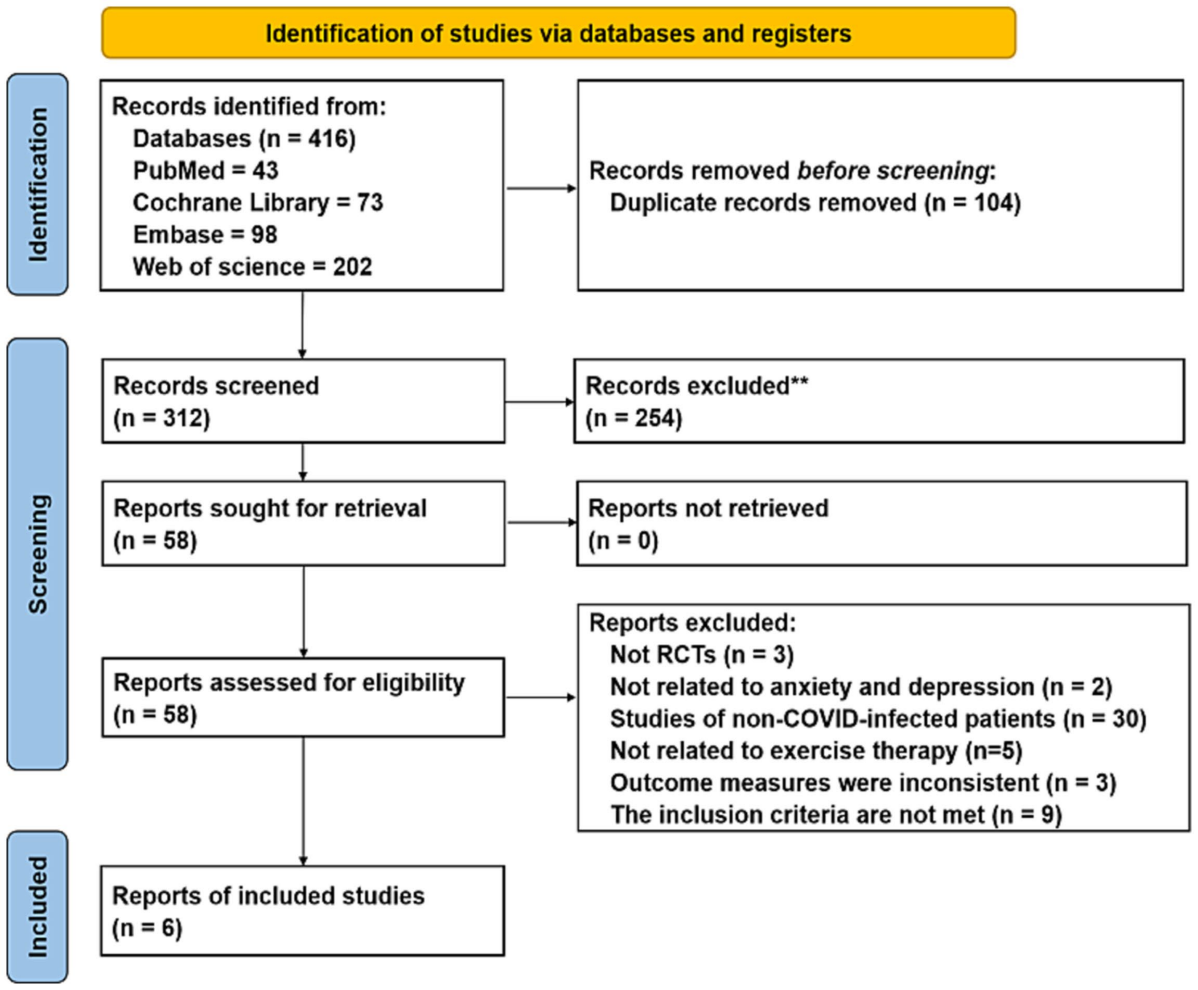
Literature screening process.

4 electronic databases were searched, yielding a total of 416 documents. 104 duplicate articles were eliminated. The titles and abstracts of these papers led to the exclusion of 294 citations. 12 researches were eliminated due to their failure to meet the qualifying requirements, leaving 18 papers that passed the full-text examination. Figure 1 illustrates how the remaining 6 RCTs, which had 461 individuals, fulfilled the eligibility requirements. Table 1 provides a summary of the baseline characteristics of the six trials.

**Table 1:** Basic characteristics of the included literature.

Study	Country	Design	Sample size	Age (mean ± SD)		Intervention time	Outcome
				Experiment	Control		
Liu et al. (16)	China	RCT	72	69.4 ± 8.0	68.9 ± 7.6	6 weeks	DLCO 6-min walk distance test QoL (SF-36) ADL SAS SDS
Bhanda-ri et al. (17)	India	RCT	38	Male: 29.7 ± 6.2 Female: 34.1 ± 15.1	Male: 34.4 ± 11 Female: 28.6 ± 12	1 month	BDI HAM-A WHO-QoL-BREF DTS
Jung et al. (18)	Korea	RCT	109	51.06 ± 16.42	45.96 ± 17.20	7 days	SAS SDS PHQ-9 VAS ISI-K
Liu et al. (19)	China	RCT	140	NA	NA	1 month	SAI PSQI
Sharma et al. (20)	India	RCT	62	49.41 ± 12.51	53.64 ± 11.55	5–18 days	HADS GAD-7 PHQ-9 PSS-10
Zhang et al. (21)	China	RCT	40	41.30 ± 7.73	42.10 ± 8.47	3 month	SAS SDS PSQI

### Risk of bias assessment

**Figure 2:**
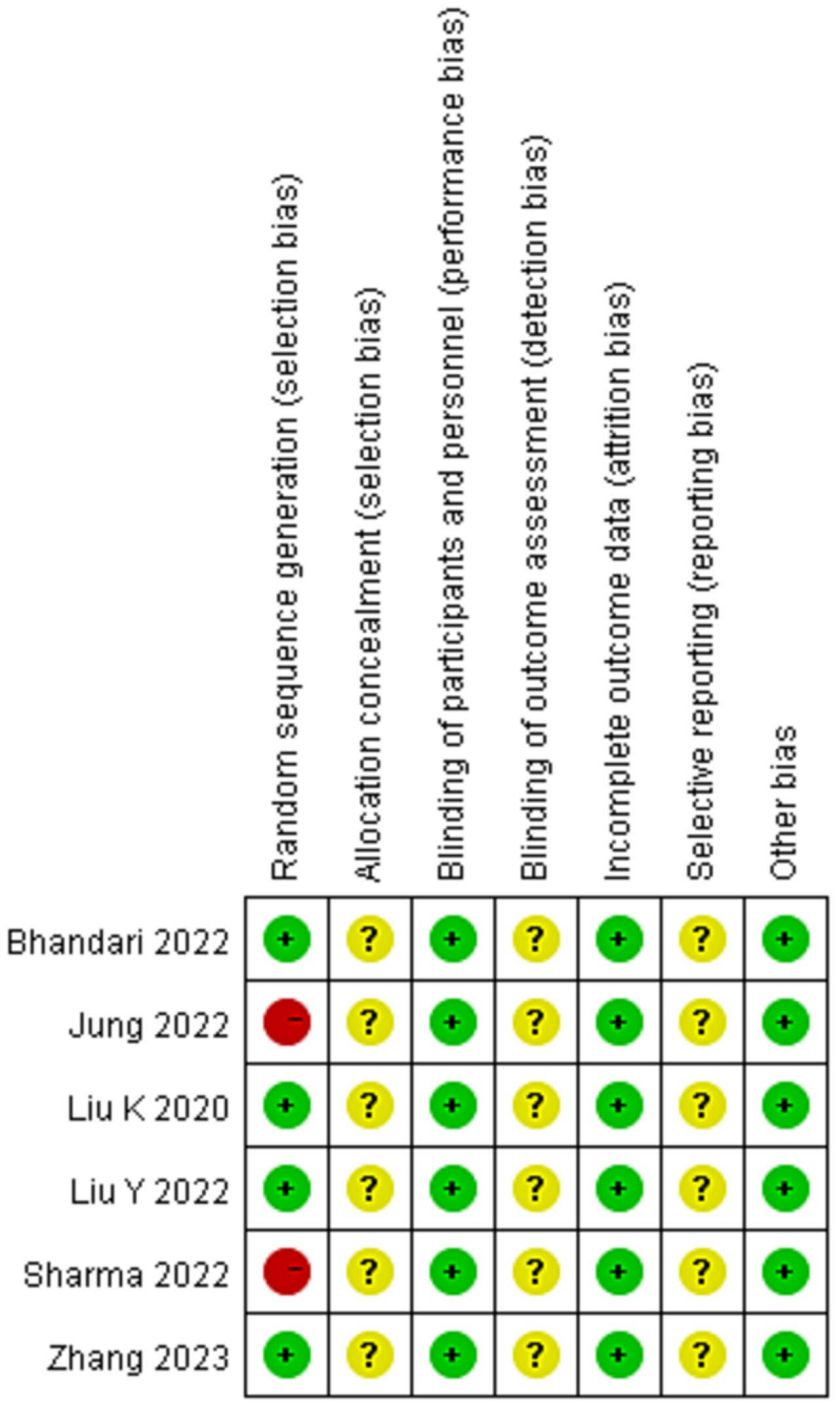
Risk of bias summary about each risk of bias item for each included study.

**Figure 3:**
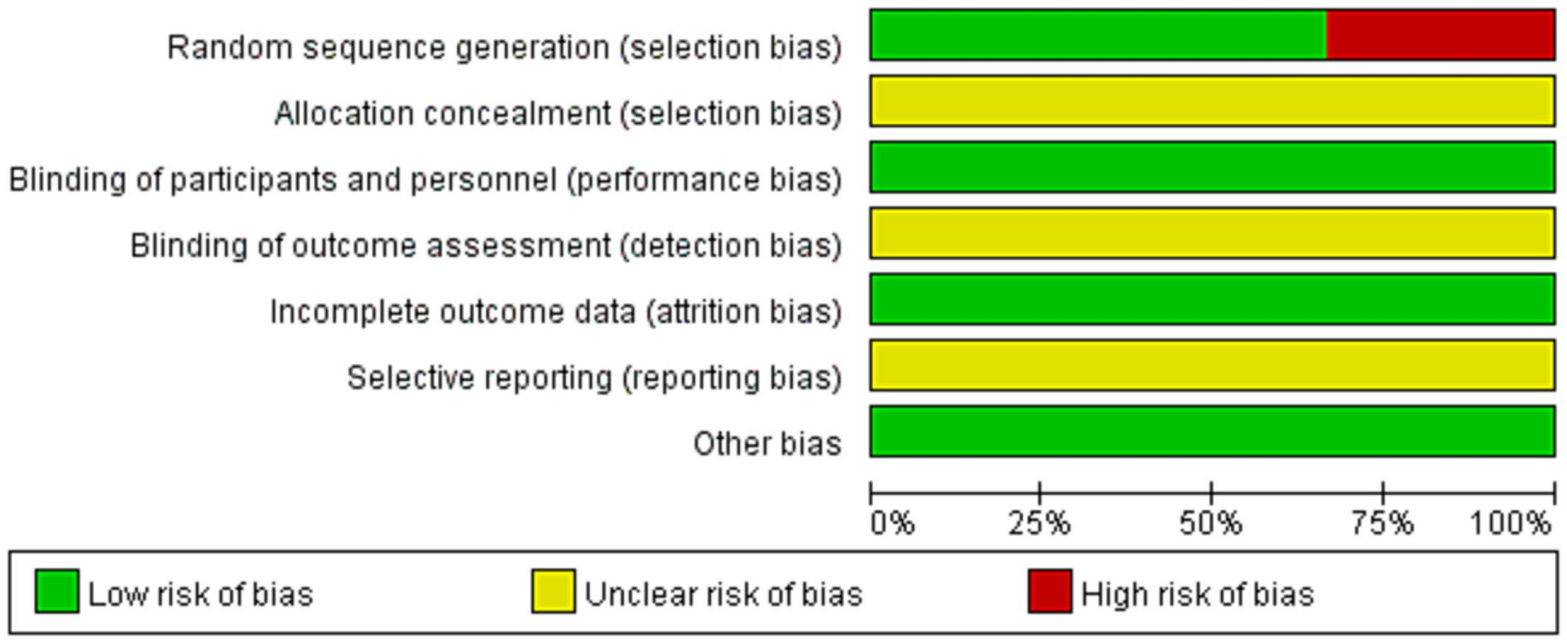
Demonstrate the risk of bias in the included studies.

All studies (16–21) mentioned randomly divided, due to Jung et al. (18) divided groups according to their bed number and Sharma’s study was a quasi-randomized study were assessed high risk of bias. None of the studies had sufficient information to judge whether to perform allocation scheme concealment and blinding of outcome evaluators were unclear risk of bias. Included studies were treated for exercise therapy, and for RCTs, the outcome indicators were not affected by allocation concealment, so all studies were low risk of bias. The outcome data for all study were complete were low risk. None of all studies had sufficient information to assess the risk of selective reporting were unclear risk of bias. No other potential sources of bias in all studies were low risk of bias (show in Figures 2, 3).

### Primary outcomes

#### Anxiety level

**Figure 4:**
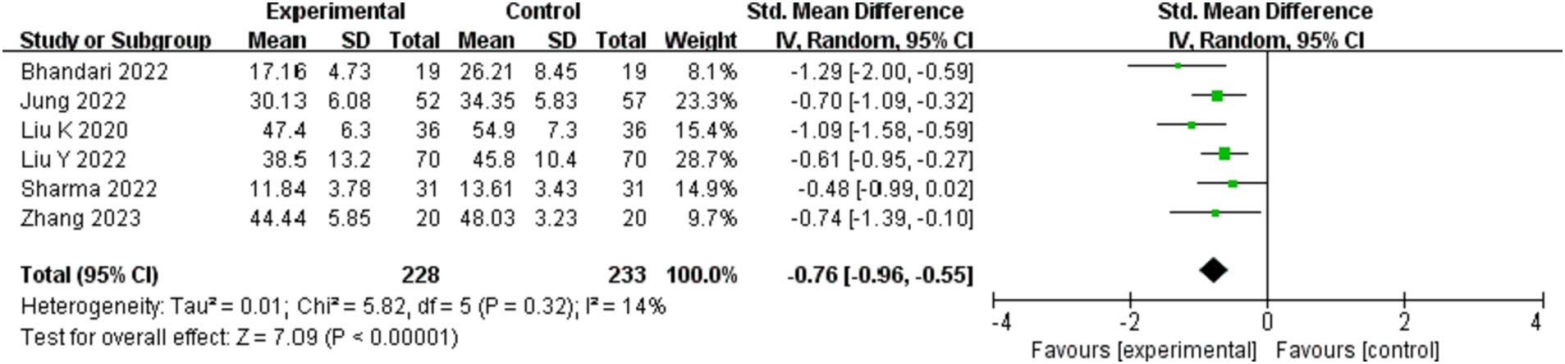
Effect of exercise therapy on anxiety in patients with COVID-19.

Six studies (16–21) involving 461 participants examined how exercise therapy affected COVID-19 patients’ anxiety. Anxiety was measured using the SAS, HAM-A, SAI, HADS, and GAD-7. Due to the wide range of dimensions, the study employed SMD to aggregate the data on anxiety symptoms. The meta-analysis shows that, in comparison to the control group, the intervention group significantly improved the anxiety levels of COVID-19 patients overall (SMD = −0.76; 95%CI: −0.96, −0.55; *p* < 0.00001), the difference was statistically significant (show in Figure 4).

### Depression level

**Figure 5:**
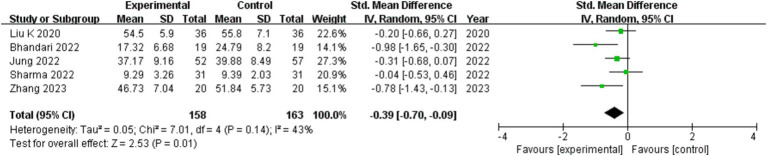
Effect of exercise therapy on depression in patients with COVID-19.

321 participants in five studies (16–18, 20, 21) assessed how exercise therapy affected COVID-19 patients’ depression. The investigation employed SMD to compile the data on depression symptoms using assessment techniques such as SDS, BDI, and HADS. The comprehensive data showed that, overall, the treatment group outperformed the control group in improving COVID-19 patient depression symptoms (SMD = −0.39; 95%CI: −0.70, −0.09; *p* = 0.01), the difference was statistically significant (show in Figure 5).

### Subgroup analysis

**Figure 6:**
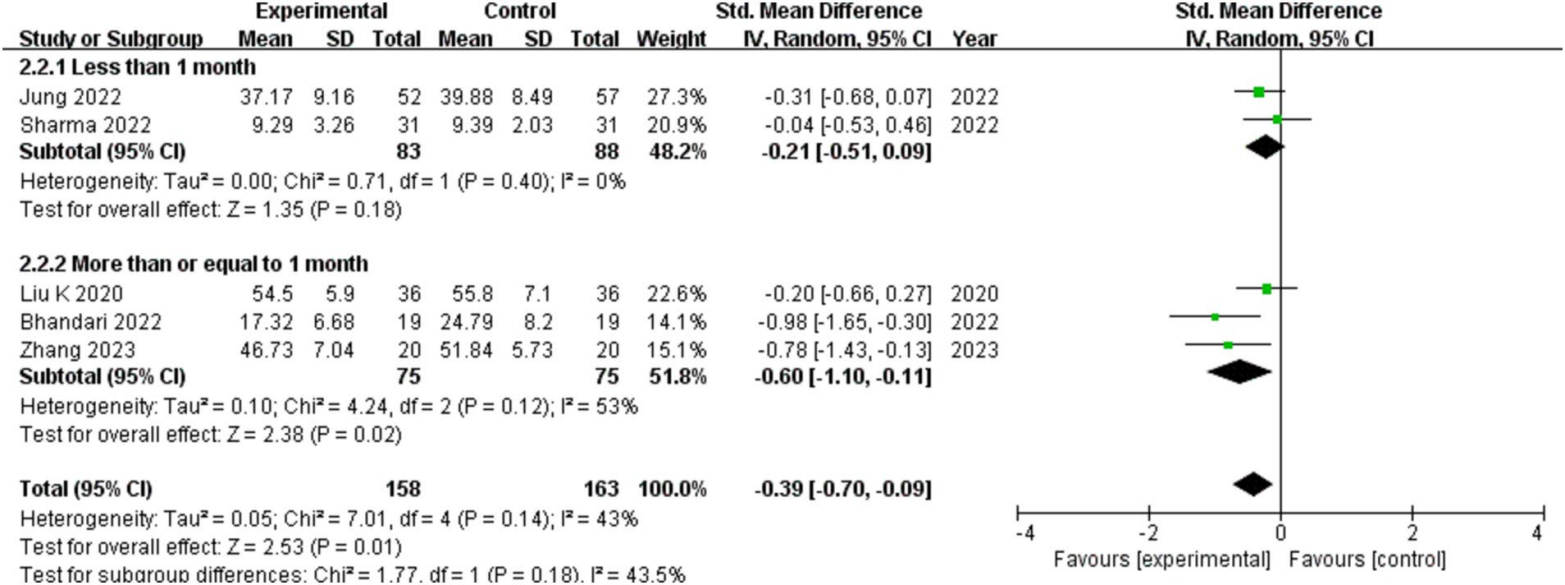
The effect of different of the length of intervention on depression in patients with COVID-19.

The treatment time is split into two categories: less than 1 month and more than 1 month, depending on how long the intervention is. The treatment group and the control group differ from one another, as seen by the results from different intervention durations. Subgroup distinctions exist, and in contrast to the control group, individuals with COVID-19 can experience improved depression with more than a month of exercise therapy (show in Figure 6).

**Figure 7:**
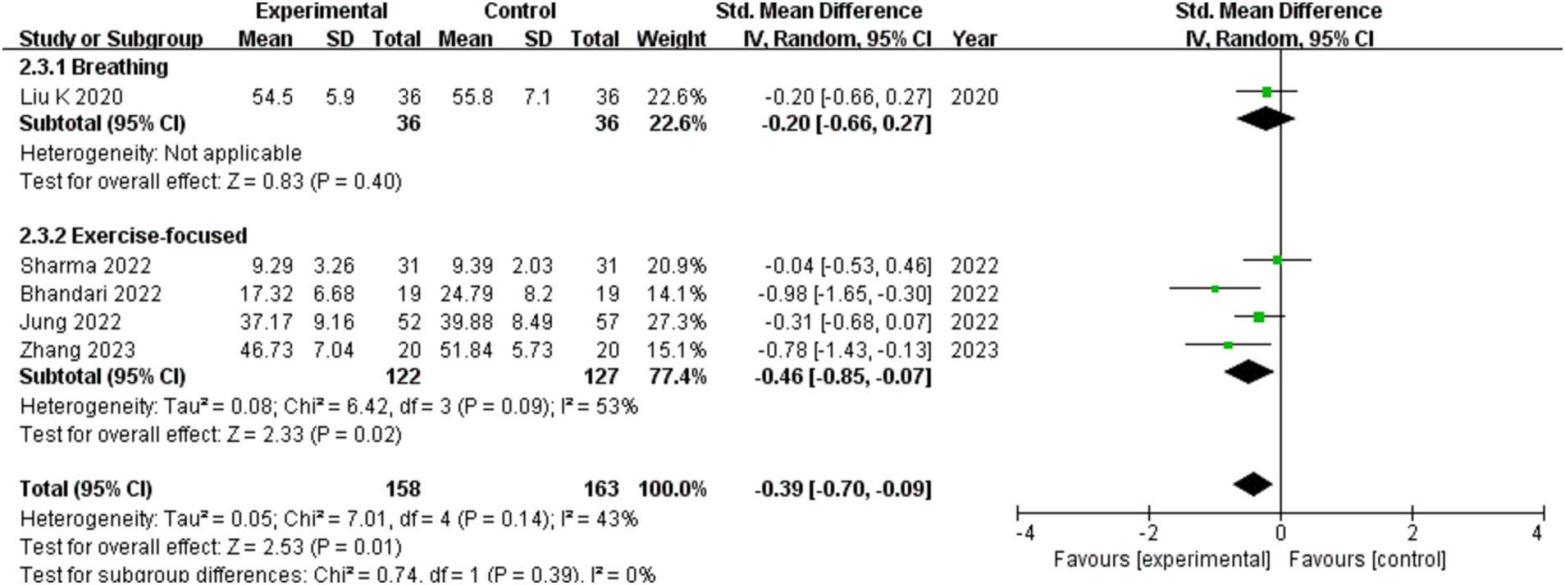
The effect of different exercise types on depression in patients with COVID-19.

One on respiratory motion study reached no conclusions. Four exercise-focused studies suggesting that there are subgroup differences, and exercise-focused exercise therapy can help COVID-19 patients who are depressed compared with the control group. Comprehensive outcomes of various intervention types demonstrate that the experimental group and the control group differ from one another (show in Figure 7).

### Secondary outcomes

#### PHQ-9

**Figure 8:**
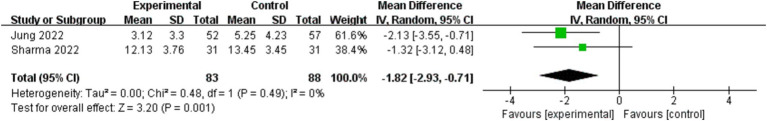
Effect of exercise therapy on PHQ-9 in patients with COVID-19.

According to DSM-IV criteria, the PHQ-9 is a standard screening tool used to identify depression. It is an accurate and reliable indicator of the severity of depression (22). The intended outcome, PHQ-9, was reported in two studies (18, 20). The intervention group significantly outperformed the control group in terms of improving PHQ-9 score (MD = −1.82; 95%CI: −2.93, −0.71; *p* = 0.001), a statistically significant difference was present (show in Figure 8).

### Quality of sleep

**Figure 9:**
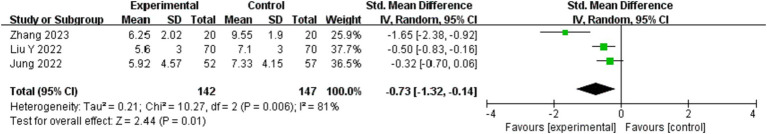
Effect of exercise therapy on the quality of sleep in patients with COVID-19.

There were 4 studies that reported on the patients’ sleep quality, and since the data from 1 study was insufficient, 3 studies (18, 19, 21) were included. Quality of sleep was assessed by the PSQI and ISI-K. The data on depressive symptoms were combined by the study using SMD. The findings indicate a noteworthy distinction in the improvement of sleep quality between the intervention group and the control group (SMD = −0.73; 95%CI: −1.32, −0.14; *p* = 0.01), the difference was statistically significant (show in Figure 9).

## Conclusion

For this study, the effects of exercise therapy on depression and anxiety were evaluated in six trials with a total of 461 COVID-19 participants. When compared to the control group, the intervention group significantly improved COVID-19 patients’ anxiety overall. When compared to the control group, the intervention group showed improvements in the depressed symptoms of COVID-19 patients. This study conducted subgroup analysis because the total results were quite statistically diverse. The findings indicated that depression could be improved with more than a month of exercise therapy, and that depression could be improved with activity-focused exercise therapy when compared to the control group in COVID-19 patients. Patients with COVID-19 may have improvements in their PHQ-9 and sleep quality with exercise therapy. In conclusion, our study discovered evidence to suggest that exercise therapy significantly improves anxiety and depression in COVID-19 patients.

The form of exercise in this study had an impact on exercise therapy to treat depression in patients, and Table 2 lists the various strategies used in each study. The impact of breathing-based exercise on depression in COVID-19 patients did not differ statistically; all other exercise modalities effectively reduced depressive symptoms in these individuals. Among other things, the genre of literature and the sample size of the included literature may have an impact on this finding. Second, the set of changes achieved was not necessarily entirely due to exercise because five of the six included trials were multimodal therapies with an emphasis on exercise therapy. In certain research, secondary outcome indicators for anxiety and depression may have affected the results.

**Table 2:** Characteristics of different exercise modality interventions.

Study	Interventions		Frequency	Anxiety assessment	Depression assessment
	Experiment	Control			
Liu et al. (16)	1.Respiratory muscle training; 2.Cough exercise; 3.Diaphragmatic training; 4.Stretching exercise; 5.Home exercise	Without any rehabilitation intervention	2 sessions per week for 6 weeks, once a day for 10 min	SAS	SDS
Bhanda-ri et al. (17)	1.Yoga exercises 2.Meditation	Routine care	Timing: 7:00 to 8:30 AM, 5 days a week	HAM-A	BDI
Jung et al. (18)	1.Physical activity 2.Education 3.Craft	Symptomatic treatment	20 min each day	SAS	SDS
Liu et al. (19)	1.Droup psychological intervention 2.Pulmonary rehabilitation exercises(Five-tone breathing exercises and Baduanjin exercises)	Routine care	Once a day, one day will last for about 30 min	SAI	NA
Sharma et al. (20)	Yoga exercises	Routine care	Once a day, 50 min yoga sessions	GAD-7	HADS
Zhang et al. (21)	Five-elements music therapy Baduanjin Qigong	Routine care	Twice a day repeatedly in sessions of about 30 min. One concentrated exercise per week	SAS	SDS

## Discussion

Currently, there are no existing licensed anti-viral therapies, so non-pharmaceutical therapies continue to be essential for COVID-19 management (23). Increased rates of mental illness, depression, anxiety, self-harm, and suicide have been linked to the COVID-19 pandemic (24, 25). Depression and anxiety are serious public health problems. It is estimated that around 20–40% of patients who experience depressive episodes do not respond clinically to the antidepressant treatments now being used, furthermore, around half of the patients who get symptom amelioration still have persistent symptoms that impair their functioning and raise the risk of relapse (26). Furthermore, 76–85% of those suffering from mental illnesses get no therapy at all, which has a detrimental effect on the individuals, their families, and society (27). Studies have shown that mental illnesses make about 2% of all diseases worldwide. Study show that from 2010 to 2030, the estimated $2.5 trillion in treatment costs are predicted to have increased to $6 trillion (28). The prevalence of anxiety and depression has increased significantly, and new treatments are urgently needed because there is still a high failure rate in the treatment of these conditions, which poses a serious threat to people’s mental health, particularly in the context of the long-term COVID-19 (29, 30). Research on anxiety and depression in COVID-19 patients has gained international attention since these conditions have a serious negative impact on patients’ and their families’ physical and emotional well-being throughout the pandemic. Furthermore, studies have shown that the COVID-19 pandemic may promote depression, anxiety symptoms, including children and adolescents (31, 32). And all types of physical activity among children and adolescents had a precipitous decline during the COVID-19 pandemic (33).

According to this study, exercise therapy could reduce anxiety and depressive symptoms in patients with COVID-19. It is well known that physical activity is good for both physical and mental health (34). When compared to psychotherapy and medicine, exercise therapy offers numerous advantages in terms of cost, side effects, and additional health benefits. It may produce results that are similar to those of psychotherapy and pharmacology (35). A study demonstrates that engaging in sport-based activities, whether solo or in a group setting, can reduce anxiety. Moreover, a higher frequency of physical activity is linked to reduced levels of melancholy and anxiety (36).

Exercise can reduce the symptoms of anxiety and depression through a variety of mechanisms. Increased oxidative stress and inflammation have been associated with depression and anxiety disorders (37–39), glucocorticoid release, overload of immune, anabolic and cardiovascular functions and hypothalamic–pituitary–adrenal (HPA) axis dysfunction, (40, 41) imbalance of intestinal flora (42) and so on in several studies and meta-analyses. Beheshti et al. found that Lipopolysaccharide (LPS)-induced depression- and anxiety-like behaviors were linked to the oxidative damage and neuroinflammation status of braintissues and the anti-LPS effects of amino guanidine included a decrease in inflammatory cytokines, a decrease in oxidative stress and a rise in anti-inflammatory mediators (43). Angulo et al. mentioned that exercise lowers oxidative damage and chronic inflammation, boosts autophagy, and enhances mitochondrial function (44). Wang et al. study found that aerobic exercise could decrease malondialdehyde (MDA), myeloperoxidase (MPO) levels and levels of IL-1β, TNF-α, and TGF-β in mice, supported that aerobic exercise was more effective in reducing cell apoptosis, oxidative stress damage, and the inflammatory response (45). Research indicates that exercise treatment can impact the HPA axis, which is crucial for mood and cognition, and can lower levels of inflammatory cytokines (46). According to Ghannoum et al., an imbalance in the gut flora had a number of detrimental outcomes, such as imbalance of neurotransmitter levels and neuronal circuits, excessive production of proinflammatory cytokines in the immunological system, disruption of the intestinal barrier, and hyper activation of the HPA axis (47). Exercise has been demonstrated by Xia et al. to modify gut microbiota and improve malfunctioning gut-brain axis (48). Exercise increases the diversity of intestinal microorganisms, enriches good bacteria, and increases the number of butyrate-producing intestinal microbes, according to research by Du et al. These benefits are linked to improved health status (49). Overall, the effects of exercise on microbiota have been reported, mostly to improve colon health by increasing microbiota diversity and balancing the populations of harmful and helpful bacteria.

Exercise therapy can be effectively implemented in clinical practice because, according to this study, it can reduce the symptoms of anxiety and depression following COVID-19. It is also more acceptable than oral medication and aids patients’ recovery with better compliance. In addition to the COVID-19, there are many epidemics such as HIV, tuberculosis (TB) and so on, which can also promote depression, anxiety symptoms (50–52). Exercise therapy can be used in future pandemics which promote depression and anxiety symptoms.

### Limitations of the study

The limited number of publications included in this analysis is the first of its many shortcomings. Second, SMD was used in the analysis of the results due to the large number of various anxiety-depression-related rating scales that were used in the collected literature. The age in the third included study was primarily concentrated over 40 years, which may restrict the generalizability of the findings. Extensive high-quality randomized controlled trials are necessary to ascertain whether exercise therapy is beneficial in treating anxiety and depression in COVID-19 patients, especially those with varying age ranges. There is not enough information to determine how exercise treatment alone affects anxiety and depression in COVID-19 patients.

## Data availability statement

The original contributions presented in the study are included in the article/Supplementary material, further inquiries can be directed to the corresponding authors.

## Author contributions

JT: Writing – original draft. L-LC: Writing – review & editing. HZ: Writing – review & editing. PW: Writing – review & editing. FM: Writing – review & editing.
