# Supplementary material for: Effects of exercise therapy on anxiety and depression in patients with COVID-19: a systematic review and meta-analysis
Source: Front Public Health. 2024 Mar 6;12:1330521. doi: 10.3389/fpubh.2024.1330521 (PMC10950912; doi:10.3389/fpubh.2024.1330521)
Supplement: Supplementary file 1 [file Table_1.pdf]

Supplementary Material

Table of contents

1. **PRISMA 2020 checklist** 2

2. **Supplementary File S1. Search strategies for databases.** 6

1. PRISMA 2020 checklist

Section and Topic	Item #	Checklist item	Location where item is reported
**TITLE**			
Title	1	Identify the report as a systematic review.	P1
**ABSTRACT**			
Abstract	2	See the PRISMA 2020 for Abstracts checklist.	P1
**INTRODUCTION**			
Rationale	3	Describe the rationale for the review in the context of existing knowledge.	P1-2
Objectives	4	Provide an explicit statement of the objective(s) or question(s) the review addresses.	P1-2
**METHODS**			
Eligibility criteria	5	Specify the inclusion and exclusion criteria for the review and how studies were grouped for the syntheses.	P2
Information sources	6	Specify all databases, registers, websites, organisations, reference lists and other sources searched or consulted to identify studies. Specify the date when each source was last searched or consulted.	P2
Search strategy	7	Present the full search strategies for all databases, registers and websites, including any filters and limits used.	Supplementary material
Selection process	8	Specify the methods used to decide whether a study met the inclusion criteria of the review, including how many reviewers screened each record and each report retrieved, whether they worked independently, and if applicable, details of automation tools used in the process.	P2
Data collection process	9	Specify the methods used to collect data from reports, including how many reviewers collected data from each report, whether they worked independently, any processes for obtaining or confirming data from study investigators, and if applicable, details of automation tools used in the process.	P2-3
Data items	10a	List and define all outcomes for which data were sought. Specify whether all results that were compatible with each outcome domain in each study were sought (e.g. for all measures, time points, analyses), and if not, the methods used to decide which results to collect.	P3
	10b	List and define all other variables for which data were sought (e.g. participant and intervention characteristics, funding sources). Describe any assumptions made about any missing or unclear information.	P3

Section and Topic	Item #	Checklist item	Location where item is reported
Study risk of bias assessment	11	Specify the methods used to assess risk of bias in the included studies, including details of the tool(s) used, how many reviewers assessed each study and whether they worked independently, and if applicable, details of automation tools used in the process.	P3
Effect measures	12	Specify for each outcome the effect measure(s) (e.g. risk ratio, mean difference) used in the synthesis or presentation of results.	P3
Synthesis methods	13a	Describe the processes used to decide which studies were eligible for each synthesis (e.g. tabulating the study intervention characteristics and comparing against the planned groups for each synthesis (item #5)).	P4
	13b	Describe any methods required to prepare the data for presentation or synthesis, such as handling of missing summary statistics, or data conversions.	P3
	13c	Describe any methods used to tabulate or visually display results of individual studies and syntheses.	P3
	13d	Describe any methods used to synthesize results and provide a rationale for the choice(s). If meta-analysis was performed, describe the model(s), method(s) to identify the presence and extent of statistical heterogeneity, and software package(s) used.	P3
	13e	Describe any methods used to explore possible causes of heterogeneity among study results (e.g. subgroup analysis, meta-regression).	P3
	13f	Describe any sensitivity analyses conducted to assess robustness of the synthesized results.	NA
Reporting bias assessment	14	Describe any methods used to assess risk of bias due to missing results in a synthesis (arising from reporting biases).	P3
Certainty assessment	15	Describe any methods used to assess certainty (or confidence) in the body of evidence for an outcome.	P3
**RESULTS**			
Study selection	16a	Describe the results of the search and selection process, from the number of records identified in the search to the number of studies included in the review, ideally using a flow diagram.	P3
	16b	Cite studies that might appear to meet the inclusion criteria, but which were excluded, and explain why they were excluded.	P3
Study characteristics	17	Cite each included study and present its characteristics.	P4

Section and Topic	Item #	Checklist item	Location where item is reported
Risk of bias in studies	18	Present assessments of risk of bias for each included study.	P3-6
Results of individual studies	19	For all outcomes, present, for each study: (a) summary statistics for each group (where appropriate) and (b) an effect estimate and its precision (e.g. confidence/credible interval), ideally using structured tables or plots.	P4-8
Results of syntheses	20a	For each synthesis, briefly summarise the characteristics and risk of bias among contributing studies.	P4-8
	20b	Present results of all statistical syntheses conducted. If meta-analysis was done, present for each the summary estimate and its precision (e.g. confidence/credible interval) and measures of statistical heterogeneity. If comparing groups, describe the direction of the effect.	P4-8
	20c	Present results of all investigations of possible causes of heterogeneity among study results.	P4-8
	20d	Present results of all sensitivity analyses conducted to assess the robustness of the synthesized results.	NA
Reporting biases	21	Present assessments of risk of bias due to missing results (arising from reporting biases) for each synthesis assessed.	P4-8
Certainty of evidence	22	Present assessments of certainty (or confidence) in the body of evidence for each outcome assessed.	P4-8
**DISCUSSION**			
Discussion	23a	Provide a general interpretation of the results in the context of other evidence.	P5
	23b	Discuss any limitations of the evidence included in the review.	P8-9
	23c	Discuss any limitations of the review processes used.	P5
	23d	Discuss implications of the results for practice, policy, and future research.	P5-8
**OTHER INFORMATION**			
Registration and protocol	24a	Provide registration information for the review, including register name and registration number, or state that the review was not registered.	P1
	24b	Indicate where the review protocol can be accessed, or state that a protocol was not prepared.	P2
	24c	Describe and explain any amendments to information provided at registration or in the protocol.	P2
Support	25	Describe sources of financial or non-financial support for the review, and the role of the funders or sponsors in the	P9

Section and Topic	Item #	Checklist item	Location where item is reported
		review.	
Competing interests	26	Declare any competing interests of review authors.	P9
Availability of data, code and other materials	27	Report which of the following are publicly available and where they can be found: template data collection forms; data extracted from included studies; data used for all analyses; analytic code; any other materials used in the review.	P9

From: Page MJ, McKenzie JE, Bossuyt PM, Boutron I, Hoffmann TC, Mulrow CD, et al. The PRISMA 2020 statement: an updated guideline for reporting systematic reviews. BMJ 2021;372:n71. doi: 10.1136/bmj.n71

For more information, visit: http://www.prisma-statement.org/

2. Supplementary File S1. Search strategies for databases.

PubMed:

A total of 43 articles were retrieved from the PubMed database on April 30, 2023, Beijing time.

Search strategies:

#1:COVID-19[mh] or SARS-CoV-2 Infection[tiab] or Infection, SARS-CoV-2[tiab] or SARS CoV 2 Infection*[tiab] or 2019 Novel Coronavirus Disease[tiab] or 2019 Novel Coronavirus Infection[tiab] or 2019 ncov disease*[tiab] or Disease, 2019-nCoV[tiab] or COVID 19 Virus Infection*[tiab] or Infection, COVID-19 Virus[tiab] or Virus Infection, COVID-19[tiab] or Coronavirus Disease 2019[tiab] or Disease 2019, Coronavirus[tiab] or Coronavirus Disease 19 [tiab] or SARS Coronavirus 2 Infection[tiab] or COVID 19 Virus Disease*[tiab] or Disease, COVID-19 Virus[tiab] or Virus Disease, COVID-19 [tiab] or 2019 nCoV Infection*[tiab] or Infection, 2019-nCoV[tiab] or COVID 19 Pandemic*[tiab] or Pandemic, COVID-19[tiab]
#2： Anxiety[mh] or Angst[tiab] or Social Anxiety*[tiab] or Anxiety*, Social[tiab] or Nervousness[tiab] or Anxiousness[tiab]
#3： Depressive Disorder[mh] or depression[mh] or Depressive Symptom*[tiab] or Symptom, Depressive[tiab] or Emotional Depression[tiab] or Depression, Emotional[tiab] or Depressive Disorders[tiab] or Disorder*, Depressive[tiab] or Neurosis, Depressive[tiab] or Depressive Neurosis[tiab] or Depression*, Endogenous[tiab] or Endogenous Depression*[tiab] or Depressive Syndrome*[tiab] or Syndrome*, Depressive[tiab] or Depression*, Neurotic[tiab] or Neurotic Depression*[tiab] or Melancholia*[tiab] or Unipolar Depression*[tiab] or Depression*, Unipolar[tiab]
#4： #2 or #3
#5： #1 and #4
#6： Exercise Therapy[mh] or Exercise[mh] or walking[mh] or sports[mh] or dancing[mh] or Remedial Exercise*[tiab] or Therapy*, Exercise[tiab] or Rehabilitation Exercise*[tiab] or Physical Activity*[tiab] or Physical Exercise*[tiab] or Acute Exercise*[tiab] or Exercise*, Isometric[tiab] or Isometric Exercise*[tiab] or Aerobic Exercise*[tiab] or Exercise Training*[tiab] or sport*[tiab] or movement therap*[tiab] or stretching[tiab] or dancing[tiab] or "Tai Ji"[tiab] or "Tai Chi"[tiab] or "Tai-Ji"[tiab] or "Tai-Chi"[tiab] or walking[tiab] or yoga[tiab] or yogic[tiab] or asana[tiab] or pranayama[tiab] or Cycle[tiab] or walk[tiab] or Treadmill[tiab]
#7： Randomized controlled trial[pt] OR controlled clinical trial[pt] or randomized[tiab] or randomly[tiab]
#8： #5 and #6 and #7

Cochrane Library:

A total of 73 articles were retrieved from the Cochrane Library database on May 1, 2023, Beijing time.

Search strategies:

#1:MeSH descriptor: [COVID-19] explode all trees
#2:("SARS-CoV-2 Infection" OR " Infection, SARS-CoV-2" OR " SARS CoV 2 Infection" OR "2019 Novel Coronavirus Disease" OR "2019 Novel Coronavirus Infection " OR "2019 ncov disease" OR " Disease, 2019-nCoV" OR " COVID 19 Virus Infection" OR " Infection, COVID-19 Virus" OR " Virus Infection, COVID-19" OR " Coronavirus Disease 2019" OR " Disease 2019, Coronavirus" OR " Coronavirus Disease 19" OR " SARS Coronavirus 2 Infection" OR " COVID 19 Virus Disease" OR " Disease, COVID-19 Virus" OR " Virus Disease, COVID-19" OR "2019 nCoV Infection" OR " Infection, 2019-nCoV" OR " COVID 19 Pandemic" OR " Pandemic, COVID-19"):ti,ab,kw
#3: #1 OR #2
#4: MeSH descriptor: [Mental Disorders] explode all trees
#5: MeSH descriptor: [Anxiety] explode all trees
#6: MeSH descriptor: [Depressive Disorder] explode all trees
#7: MeSH descriptor: [depression] explode all trees
#8: ("Mental Disorder" OR "Psychiatric Illness" OR "Psychiatric Disease" OR "Mental Illness" OR "Illness, Mental" OR "Psychiatric Disorder" OR "Diagnosis,Psychiatric" OR "Mental Disorder, Severe" OR "Psychiatric Diagnosis" OR "Severe Mental Disorder" OR "Angst" OR "Nervousness" OR "Anxiousness" OR "Socialanxiet" OR "Disorder, Depressive" OR "Neurosis, Depressive" OR "Depressive Neurosis" OR "Depression, Endogenous" OR "Endogenous Depression" O"Depressive Syndrome" OR "Neurotic Depression" OR "Melancholia" OR "Unipolar Depression" OR "Depressive Symptom" OR "Depression, Emotional"):ti,ab,kw
#9: #4 OR #5 OR #6 OR #7 OR #8
#10: MeSH descriptor: [Exercise] explode all trees
#11: MeSH descriptor: [Exercise Therapy] explode all trees
#12: MeSH descriptor: [walking] explode all trees
#13: MeSH descriptor: [sports] explode all trees
#14: MeSH descriptor: [dancing] explode all trees
#15: ("Therapy, Exercise" OR "Rehabilitation Exercise" OR "Physical Activity" OR "Physical Exercise" OR "Acute Exercise" OR "Exercise, Isometric" OR "Isometric Exercise" OR "Aerobic Exercise" OR "Exercise Training" OR "Interval training" OR "sport" OR "movement therapy" OR "stretching" OR "dancing" OR "Tai Ji" OR "Tai Chi" OR "Tai Chi" OR "Tai-Chi" OR "walking" OR "yoga" OR "yogic" OR "asana" OR "pranayama" OR "Cycle" OR "walk" OR "Treadmill"):ti,ab,kw
#16: #10 OR #11 OR #12 OR #13 OR #14 OR #15
#17: MeSH descriptor: [Randomized Controlled Trial] explode all trees
#18: MeSH descriptor: [Clinical Study] explode all trees
#19: MeSH descriptor: [Clinical Trial] explode all trees
#20: MeSH descriptor: [Controlled Clinical Trial] explode all trees
#21: MeSH descriptor: [Comparative Study] explode all trees
#22: MeSH descriptor: [Equivalence Trial] explode all trees
#23: MeSH descriptor: [Clinical Studies as Topic] explode all trees
#24: MeSH descriptor: [Clinical Trials as Topic] explode all trees

#25: MeSH descriptor: [Controlled Clinical Trials as Topic] explode all trees
#26: MeSH descriptor: [Equivalence Trials as Topic] explode all trees
#27: MeSH descriptor: [Pragmatic Clinical Trials as Topic] explode all trees
#28: MeSH descriptor: [Case-Control Studies] explode all trees
#29: MeSH descriptor: [Random Allocation] explode all trees
#30: ("randomized controlled trial" OR "Clinical Study" OR "Clinical trial" OR "controlled clinical trial" OR "comparative study" OR "Equivalence Trial" OR "Pragmatic clinical trial" OR "Clinical Trials, Randomized" OR "Trials, Randomized Clinical" OR "Controlled Clinical Trials, Randomized" OR "Clinical Trial as Topic" OR "Comparative Studies" OR "Naturalistic Randomized Clinical Trial" OR "Practical Clinical Trials" OR "Clinical Trials, Practical" OR "Pragmatic Trials" OR "Trials, Pragmatic" OR "Pragmatic Clinical Trials" OR "Clinical Trials, Pragmatic" OR "Trials, Pragmatic Clinical" OR "Clinical study" OR "Clinical trial" OR "Pragmatic clinical trial" OR "Allocation, Random" OR "Randomization" OR "Randomized" OR "Placebo" OR "randomly" OR "trial" OR "Case-control" OR "Case-Control Study" OR "Studies, Case-Control" OR "Study, Case-Control" OR "Case-Comparison Studies" OR "Case-Comparison Study" OR "Case-Compeer Studies" OR "Case-Base Studies" OR "Case Base Studies" OR "Studies, Case-Base" OR "Case Control Studies" OR "Case Control Study" OR "Studies, Case Control" OR "Study, Case Control" OR "comparative study" OR "risk factors" OR "case control"):ti,ab,kw
#31: #17 OR #18 OR #19 OR #20 OR #21 OR #22 OR #23 OR #24 OR #25 OR #26 OR #27 OR #28 OR #29 OR #30
#32: #3 AND #9 AND #16 AND #31

Embase

A total of 98 articles were retrieved from Embase database on May 1, 2023, Beijing time.

Search strategies:

#1: 'COVID-19'/exp OR 'SARS-CoV-2 Infection':ab,ti OR 'Infection, SARS-CoV-2':ab,ti OR 'sars cov 2 infection?':ab,ti OR '2019 Novel Coronavirus Disease':ab,ti OR '2019 Novel Coronavirus Infection':ab,ti OR '2019 ncov disease?':ab,ti OR 'Disease, 2019-nCoV':ab,ti OR 'COVID 19 Virus Infection?':ab,ti OR 'Infection, COVID-19 Virus':ab,ti OR 'Virus Infection, COVID-19':ab,ti OR 'Coronavirus Disease 2019':ab,ti OR 'Disease 2019, Coronavirus':ab,ti OR 'Coronavirus Disease 19':ab,ti OR 'SARS Coronavirus 2 Infection':ab,ti OR 'COVID 19 Virus Disease?':ab,ti OR 'Disease, COVID-19 Virus':ab,ti OR 'Virus Disease, COVID-19':ab,ti OR '2019 nCoV Infection?':ab,ti OR 'Infection, 2019-nCoV':ab,ti OR 'COVID 19 Pandemic?':ab,ti OR 'Pandemic, COVID-19':ab,ti
#2: 'Mental Disorders'/exp OR 'Anxiety'/exp OR 'depression'/exp OR 'Depressive Disorder'/exp

#3: 'Mental Disorder':ab,ti OR 'Psychiatric Illness':ab,ti OR 'Psychiatric Disease?':ab,ti OR 'Mental Illness':ab,ti OR 'Illness, Mental':ab,ti OR 'Psychiatric Disorder?':ab,ti OR 'Diagnosis, Psychiatric':ab,ti OR 'Psychiatric Diagnosis':ab,ti OR 'Mental Disorder?,Severe':ab,ti OR 'Severe Mental Disorder?':ab,ti OR 'Angst':ab,ti OR 'Nervousness':ab,ti OR 'Anxiousness':ab,ti OR 'Social Anxiet*':ab,ti OR 'Anxiet*,Social':ab,ti OR 'Depressive Disorders':ab,ti OR 'Disorder?, Depressive':ab,ti OR 'Neurosis, Depressive':ab,ti OR 'Depressive Neurosis':ab,ti OR 'Depression?,Endogenous':ab,ti OR 'Endogenous Depression?':ab,ti OR 'Depressive Syndrome':ab,ti OR 'Depression, Neurotic':ab,ti OR 'Melancholia':ab,ti OR 'Unipolar Depression':ab,ti OR 'Depressive Symptom':ab,ti OR 'Emotional Depression':ab,ti
#4: #2 OR #3
#5: #1 AND #4
#6: 'ExerciseTherapy'/exp OR 'Exercise'/exp OR 'walking'/exp OR 'sports'/exp OR 'dancing'/exp OR 'Therap*,Exercise':ab,ti OR 'RehabilitationExercise?':ab,ti OR 'Physicalactivity':ab,ti OR 'PhysicalExercise?':ab,ti OR 'AcuteExercise?':ab,ti OR 'AerobicExercise?':ab,ti OR 'ExerciseTraining':ab,ti
#7: 'Interval training':ab,ti OR 'sport':ab,ti OR 'movement therap*':ab,ti OR 'stretching':ab,ti OR 'dancing':ab,ti OR 'Tai Ji':ab,ti OR 'Tai Chi':ab,ti OR 'Tai-Ji':ab,ti OR 'walking':ab,ti OR 'yoga':ab,ti OR 'yogic':ab,ti OR 'asana':ab,ti OR 'pranayama':ab,ti OR 'Cycle':ab,ti OR 'walk':ab,ti OR 'Treadmill':ab,ti
#8: #6 or #7
#9: 'randomized controlled trial'/exp OR 'Clinical Study'/exp OR 'Clinical trial'/exp OR 'controlled clinical trial'/exp OR 'Case Control Study'/exp OR 'comparative study'/exp OR 'Equivalence Trial'/exp OR 'Pragmatic clinical trial':ab,ti OR 'randomized controlled trial (topic)'/exp OR 'clinical trials (topic)'/exp OR 'controlled clinical trial (topic)'/exp OR 'Equivalence Trial (topic)'/exp OR 'Clinical Trial$, Randomized':ab,ti OR 'Trial$, Randomized Clinical':ab,ti OR 'Comparative Stud*':ab,ti OR 'Practical Clinical Trial$':ab,ti OR 'Clinical Trial$, Practical':ab,ti OR 'Trial$, Practical Clinical':ab,ti OR 'Pragmatic Trial$':ab,ti OR 'Trial$, Pragmatic':ab,ti OR 'Pragmatic Clinical Trial$':ab,ti OR 'Random allocation':ab,ti OR 'Allocation,Random':ab,ti OR 'Randomization'/exp OR 'Random*':ab,ti OR 'Placebo':ab,ti OR

'trial$':ab,ti OR 'Case-control':ab,ti OR 'Case Control':ab,ti

#10: #5 AND #8 AND #9 AND ([article]/lim OR [article in press]/lim) AND [humans]/lim AND [clinical study]/lim AND ([controlled clinical trial]/lim OR [randomized controlled trial]/lim)

Web of science

A total of 202 articles were retrieved from Embase database on May 1, 2023, Beijing time.

Search strategies:

TS=(COVID-19 OR SARS-CoV-2 Infection OR 2019 Novel Coronavirus Disease OR 2019 nCoV Disease OR 2019-nCoV Diseases OR COVID-19 Virus Infection OR Coronavirus Disease 2019 OR SARS Coronavirus 2 InfectionORCOVID-19 Virus Disease OR 2019-nCoV Infection OR COVID-19 Pandemic)AND TS=(Anxiety OR Anxiety disorders OR Depressive Disorder OR depression)AND TS=(Exercise Therapy OR Exercise OR walking OR sports OR Rehabilitation Exercise OR Physical activity OR Physical Exercise OR Aerobic Exercise OR Exercise Training OR movement therapy OR Tai Ji OR Tai Chi OR Tai-Ji OR Tai-Chi OR yoga OR yogic) AND TS=(Randomized controlled trial OR RCT)
